# Discovery of Nobiletin from Citrus Peel as a Potent Inhibitor of β-Amyloid Peptide Toxicity

**DOI:** 10.3390/nu11112648

**Published:** 2019-11-04

**Authors:** Kumju Youn, Seonah Lee, Mira Jun

**Affiliations:** 1 Department of Food Science and Nutrition, College of Health Sciences, Dong-A University, 37, Nakdong-daero 550 beon-gil, Saha-gu, Busan 49315, Korea; kjyoun@dau.ac.kr (K.Y.); seonah_lee@daum.net (S.L.); 2 Center for Silver-Targeted Biomaterials, Brain Busan 21 Plus Program, Graduate School, Dong-A University, 37, Nakdong-daero 550 beon-gil, Saha-gu, Busan 49315, Korea

**Keywords:** Alzheimer’s disease, amyloid-β peptide, oxidative stress, inflammation, nobiletin

## Abstract

Increasing evidence has demonstrated that amyloid-β peptide (Aβ), the hallmark of Alzheimer’s disease (AD), evokes oxidative and inflammatory cascades, which ultimately lead to the death of neurons. The purpose of the present study is to demonstrate the effect of nobiletin, a representative compound of citrus peel, in preventive and therapeutic approaches against neuronal damage by exposure to Aβ_25–35_. Nobiletin significantly ameliorated Aβ_25–35-_mediated cell death by restoring abnormal changes in intracellular oxidative stress, cell cycle, nuclear morphology, and activity of apoptotic caspase. Regarding anti-inflammatory responses, nobiletin significantly suppressed interleukin-1β, tumor necrosis factor-α, nitric oxide (NO), and prostaglandin E_2_ production in response to Aβ stimulation. Moreover, nobiletin inhibited Aβ-stimulated inducible NO synthase and cyclooxygenase-2 expression, which was attributed to the blockade of nuclear factor-κB p65 and phosphorylation of its inhibitor, IκB-α. Interestingly, nobiletin decreased expression of c-Jun N-terminal kinase and p38 without affecting extracellular signal-regulated kinase 1/2 activation. Taken together, the novel data implicate nobiletin as a potential candidate for the prevention of AD through the inhibition of oxidative stress, apoptosis, and inflammation.

## 1. Introduction

Amyloid-β peptide (Aβ) is the major component of amyloid plaque. Aβ is a characteristic marker of Alzheimer’s disease (AD) and is believed to initiate the pathological cascade of the disease. Aβ is a 39 to 43 amino acid peptide formed by β- and γ-secretases that catalyze the splitting of amyloid precursor protein (APP) [1]. Aβ accumulation triggers the cascade of events, such as reactive oxygen species (ROS) production, cell cycle dysregulation, tau phosphorylation, and inflammation, which, ultimately, lead to the death of neurons [2,3].

The detrimental role of Aβ in the stimulation of oxidative stress has been reported in various studies. In a transgenic mouse model, Aβ increased the levels of protein and lipid oxidation markers, including protein carbonyls, 4-hydroxy-2-nonenal, and 3-nitrotyrosine [4,5]. The observed oxidative injury appeared to be dependent on the methionine 35 of Aβ peptide [6]. Notably, Aβ can regulate ROS generation and ROS can reciprocally promote overproduction of Aβ in a vicious cycle [7].

Nuclear factor-kappa B (NF-κB) regulates Aβ homeostasis through transcriptional upregulation of various related enzymes and proteins [8]. Under physiological conditions, NF-κB regulates the expression rates of genes for APP, β-secretase 1 (BACE1) and several components of the γ-secretase complex. However, activation of NF-κB by Aβ regulates the transcription of all these genes and over-stimulates Aβ production [9]. Mitogen-activated protein kinases (MAPKs), such as extracellular protein regulated protein kinase (ERK), c-Jun N-terminal kinase (JNK) and p38, can activate the NF-κB pathway to produce a series of inflammatory factors.

Nobiletin (2-(3,4-dimethoxyphenyl)-5,6,7,8-tetramethoxychromen-4-one) is one of the major polymethoxyflavones in the peel of citrus fruits, including oranges, mandarins, limes, and lemons [10]. Various pharmacological effects attributed to nobiletin include antioxidant, anti-inflammatory, anti-diabetic, anti-atherogenic and anti-carcinogenic activities [11,12,13,14,15]. The neuroprotective property of nobiletin has been demonstrated in several recent studies. The compound prevents ischemic brain injury by regulating the Akt/cAMP-response element-binding protein (CREP)/Bcl-2 pathway in Sprague–Dawley rats. Nobiletin reduces Aβ-stimulated memory impairment in several AD animal models, including olfactory bulbectomy mice, APP-SL 7–5 Tg mice and 3XTg-AD mice [16,17,18].

Our previous study demonstrated the ability of nobiletin to non-competitively inhibit BACE1 via hydrogen-bond-mediated interactions with allosteric residues of the enzyme [19]. However, the primary molecular mechanism underlying the neuroprotective effect of nobiletin on Aβ-induced oxidative stress and inflammation has not yet been clearly demonstrated, which prompted the present investigation of the possible effects of nobiletin on PC12 cell models.

## 2. Materials and Methods 

### 2.1. Cell Culture and Aβ_25-35_ Stock Solution

PC12 cells were cultured in RPMI1640 medium supplemented with 10% horse serum, 5% fetal bovine serum, and penicillin (100 U/mL) and treated with nobiletin (Sigma-Aldrich, St. Louis, MO, USA) for 24 h at 37 °C in a 5% CO_2_ incubator. Aβ_25-35_ (Sigma-Aldrich) was dissolved in dimethylsulfoxide (DMSO) at an initial concentration of 10 mM and diluted with phosphate buffered saline (PBS). Aβ_25-35_ solution was incubated at 37 °C for 48 h to permit aggregation before use.

### 2.2. Cell Viability Analysis

Cell viability was assessed using the established 3-(4,5-dimethylthiazol-2-yl)-2,5-diphenyltetrazolium bromide (MTT) assay. PC12 cells (1×10^5^ cells/well in a 96-well plate) were incubated with MTT reagent for 3 h at 37 °C. The medium was removed and the formazan crystals produced by the reduction of MTT were dissolved in DMSO. The absorbance due to the formazan crystals was measured at 570 nm using a model ELX808 spectrophotometer (BioTek, Winooski, VT, USA).

### 2.3. Intracellular ROS Analysis

PC12 cells were stained with CM-H_2_DCFDA in minimum essential medium without serum at 37 °C for 30 min in the dark and resuspended in Hank’s balanced salt solution. Cells were placed on glass slides and cultured overnight. Imaging ROS was done by fluorescence spectrophotometry with excitation and emission wavelengths of 485 and 528 nm, respectively, using a model FLX800 spectrometer (BioTek).

### 2.4. Apoptosis Assay by Hoechst 33342 Staining

PC12 cells were harvested with PBS and fixed in 4% paraformaldehyde for 20 min at 25 °C and then washed with PBS before being exposed to Hoechst 33342 for 15 min in the dark. The apoptotic morphology was observed using fluorescence microscopy (Olympus Optical Co., Tokyo, Japan) at 400× magnification.

### 2.5. Fluorescence-Activated Cell Sorting (FACS) Analysis

PC12 cells were cultured in 24-well plates (5 × 10^5^ cells/well) and incubated with 50 μM Aβ_25-35_ for 24 h after pretreatment with various concentrations of nobiletin for 1 h. After incubation, cells were collected and analyzed using the Muse^TM^ Cell Analyzer (Merck Millipore, Darmstadt, Germany). Briefly, FACS analysis following staining with annexin V and 7-aminoactinomycin D (7-AAD) was performed to detect apoptosis, and cell viability was determined using a DNA-binding dye. Cells were fixed in cold 70% ethanol and stained with propidium iodide (PI), a membrane impermeant dye, to analyze the cell cycle.

### 2.6. Assessment of Levels of Nitric Oxide (NO) and Prostaglandin E2 (PGE_2_)

PC12 cells (2 × 10^6^) were seeded on 6-cell plates containing medium and incubated with Aβ_25-35_ for 24 h in the presence or absence of nobiletin. The formation of NO_2_^−^, a stable end product that has been extensively used as an indicator of NO accumulation, was assessed using Griess reagent. The media were mixed with Griess reagent at RT for 10 min and the NO levels were analyzed using the aforementioned ELX808 microplate reader at 570 nm.

The supernatants were mixed with primary antibody solution and PGE_2_ conjugate for 2 h, followed by washing and addition of stop solution. The absorbance was measured at 450 nm using the ELX808 microplate reader.

### 2.7. Western Blot Analysis

Proteins (40 μg) were separated by 10% sodium dodecyl sulfate-polyacrylamide gel electrophoresis and transferred to polyvinylidene difluoride (PVDF) membranes. The blots were blocked for 2 h at RT with 5% skim milk in 0.1% Tween 20 in PBS (PBST) and then incubated overnight with specific primary antibodies at 4 °C. The primary antibodies used were against β-actin (1:1000), tumor necrosis factor-alpha (TNF-α; 1:1000), cyclooxygenase-2 (COX-2; 1:1000), inducible NO synthase (iNOS; 1:1000), p-65 (1:1000), phospho (p)-IκB-α (1:1000), p-JNK (1:1000), p-p38 (1:1000), and p-ERK (1:1000). Subsequently, horseradish peroxidase (HRP)-conjugated anti-rabbit IgG secondary antibody or anti-goat IgG was used. Protein bands were visualized using the EZ-capture device (Atto, Tokyo, Japan).

### 2.8. Statistical Analysis

Statistical analysis was performed using SAS 9.3 software (SAS Institute Inc., Cary, NC, USA). All results are expressed as mean ± SD and are representative of the data obtained from three independent experiments. Statistical comparisons of differences between groups were performed through the Student’s *t* test, considering * *p* < 0.05, ** *p* < 0.01, and *** *p* < 0.001 as being statistically significant.

## 3. Results and Discussion

### 3.1. Nobiletin Inhibits Cytotoxicity Evoked by Aβ_25-35_

**Figure 1:**
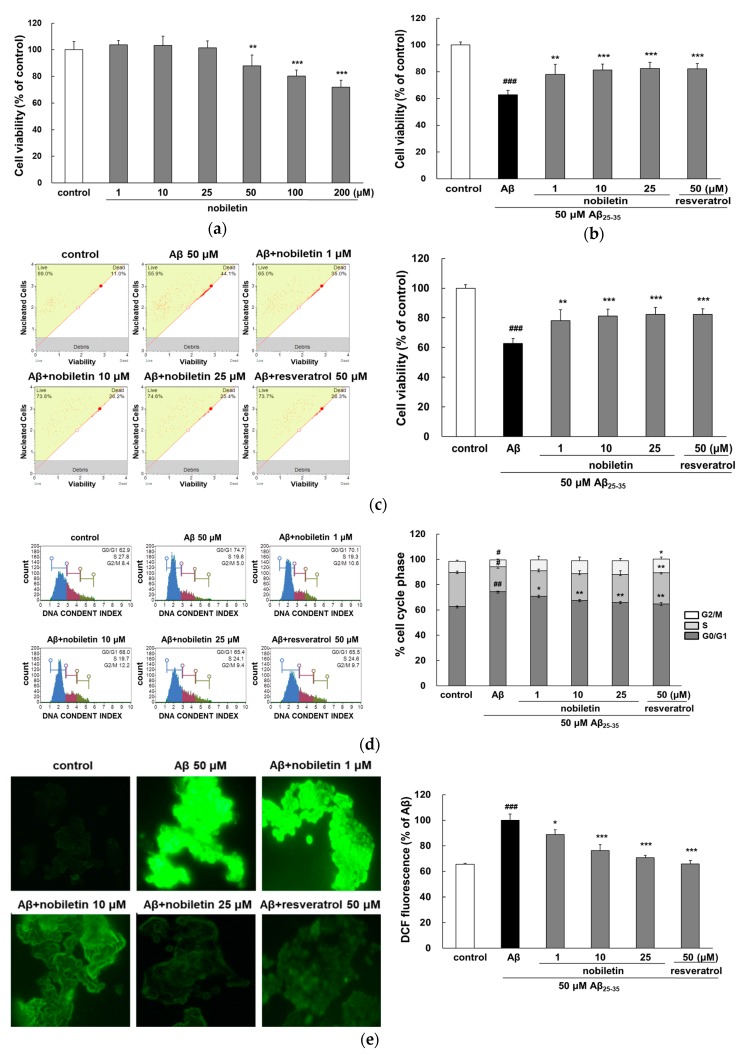
Protective properties of nobiletin against Aβ_25-35_-mediated cell damage. (**a**) Evaluation of cytotoxicity nobiletin alone in PC12 cells. Cells were pretreated with nobiletin for 1 h followed by exposure to 50 μM of Aβ_25–35_ for 24 h, and cell viability was assessed by (**b**) MTT reduction assay and (**c**) fluorescence-activated cell sorting (FACS) analysis. (**d**) Cell cycle progression was measured by FACS. The percentage of cells in the G_0_/G_0_, S, and G_0_/M phases of the cell cycle was determined using the Muse 1.5 Analysis software. (**e**) Intracellular ROS production was observed by CM-H_2_DCFDA fluorescent dye. ^###^ *p*<0.001, ^##^ *p* < 0.01, and ^#^ *p* < 0.05 vs. control. *** *p* < 0.001, ** *p* < 0.01, and * *p* < 0.05 vs. Aβ_25–35_.

To verify the neuroprotective effect of nobiletin, cell viability was assessed using the MTT assay and FACS. As shown in Figure 1a, nobiletin did not adversely affect PC12 cell viability at concentrations of 1 to 25 µM, which were used for further study. Treatment with 50 μM Aβ_25-35_ for 24 h induced approximately 40% cell death in comparison with the control group (*p* < 0.001; Figure 1b). However, pretreatment with 1, 10, and 25 μM nobiletin significantly increased cell viability up to 78.1% ± 7.4%, 81.3% ± 4.5%, and 82.4% ± 4.7%, respectively. Notably, 10 μM nobiletin exhibited a similar neuroprotective effect to that of resveratrol, a well-known positive control. Consistent with the results of the MTT assay, nobiletin significantly prevented Aβ_25-35_-induced cell death in FACS analysis (Figure 1c), and this effect was dependent on the dose of nobiletin. These data provided correlative evidence indicating that nobiletin contributes to cell survival in PC12 cells damaged by Aβ_25-35_.

Cell cycle regulation is a crucial process of cell growth and proliferation in neurons [20]. As shown in Figure 1d, Aβ_25-35_ significantly induced an increase in cells in the G_0_/G_1_ phase (*p* < 0.01) and a corresponding decrease in cells in the S phase and G_2_/M phase (*p* < 0.05), suggesting that cells had lower rates of growth and tended to be arrested at the G_0_/G_1_ transition. However, nobiletin restored Aβ_25-35_-mediated cell cycle dysregulation in a concentration-dependent manner, which may contribute to the enhanced cell viability effect of the compound.

Nobiletin was further evaluated for its antioxidant property in Aβ_25-35_-injured cells by ROS regulation. As indicated in Figure 1e, fluorescence intensity and large numbers of bright particles in cells were visibly increased by Aβ_25-35_ exposure, suggesting the presence of intracellular oxidative stress. Aβ_25-35_ stimulated significant increase in ROS to 100% ± 4.86% (*p* < 0.001). However, pretreatment of nobiletin decreased ROS generation in a dose-dependent manner (*p* < 0.05 and *p* < 0.001). These data were consistent with previous descriptions of the antioxidant property of nobiletin. The compound protected PC12 cells against H_2_O_2_-triggered damage by scavenging ROS, decreasing malonaldehyde (MDA), and enhancing glutathione (GSH) and superoxide dismutase (SOD) contents [21]. In addition, excessive intracellular ROS stimulates the activation of signal transduction cascades, which disturbs calcium homeostasis and leads to the initiation of apoptosis. Nobiletin prevents mitochondrial calcium overload as well as ROS generation in glutamate-induced cortical neurons [22]. In vivo, the intraperitoneal administration of nobiletin reportedly reduced tau phosphorylation, the index of protein oxidation, and protein carbonyl levels in SAMP8 mice, which were related to the recovery of GSH/glutathione disulfide (GSSG) ratio and increased glutathione peroxidase (GPx) activity [23]. These results demonstrated that the neuroprotective role of nobiletin results, at least in part, from the reduction of oxidative stress.

### 3.2. Nobiletin Reduces Aβ_25–35_-Mediated Apoptosis and Caspase-3 Activation

**Figure 2:**
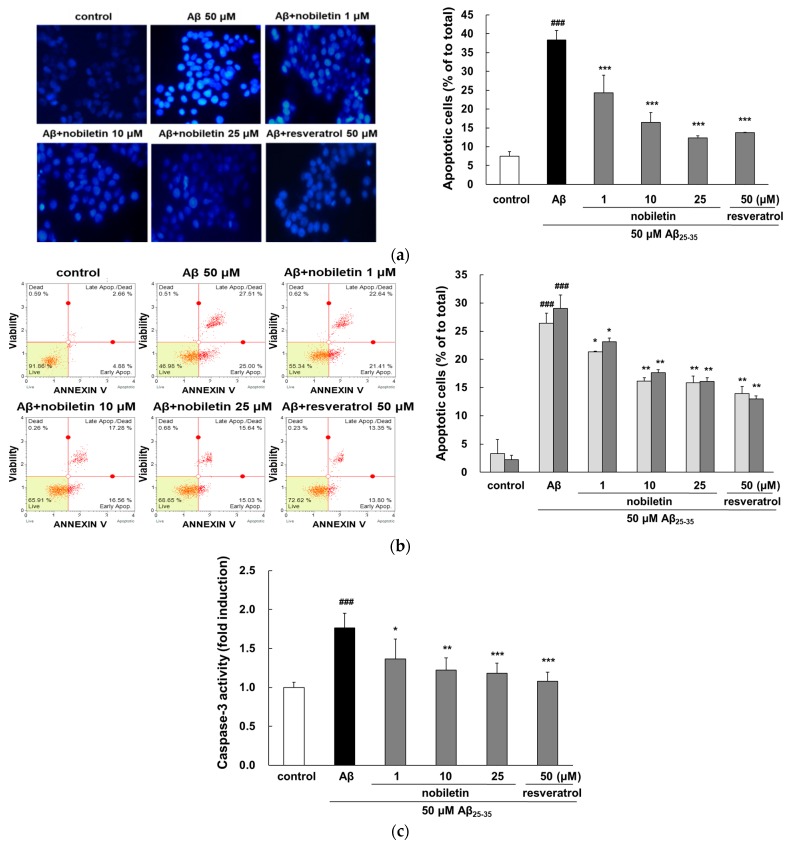
Activity of nobiletin in preventing Aβ_25-35_-evoked apoptosis and caspase-3. (**a**) Morphological features of apoptotic cells were observed by fluorescence microscopy using Hoechst 33342 staining (magnification ×400). (**b**) Flow cytometric analysis was used to investigate the properties of nobiletin on Aβ_25-35_-stimulated apoptosis. The cell populations discriminated in each quadrant are live cells in the lower-left quadrant, early apoptotic cells in the lower-right quadrant, late apoptotic cells in the upper-right quadrant, and dead cells in the upper-left quadrant. (**c**) Caspase-3 activity was assessed using the caspase-3 assay kit. ^###^ *p* < 0.001 vs. control. *** *p* < 0.001, ** *p* < 0.01, and * *p* < 0.05 vs. Aβ_25–35_.

Intracellular oxidative stress plays a central role in the induction of neuronal apoptosis stimulated by Aβ [24]. As shown in Figure 2a, cells treated with Aβ_25–35_ exhibited uneven morphology of their nuclei as a result of membrane blebbing, chromatin aggregation, and nuclear condensation, whereas the nobiletin pretreated group showed dispersed and weak fluorescence that is typical of live cells. Particularly, nobiletin at 25 μM decreased apoptosis similar to the level of the positive control group. Analysis of morphology alone is not sufficient to distinguish between early and late apoptotic cells. Thus, flow cytometric analysis was employed to quantitatively analyze apoptotic cell death. When exposed to Aβ_25-35_, early and late apoptosis was significantly increased to 26.39% ± 2.48% and 29.06% ± 2.33%, respectively, compared with the control group (*p* < 0.001, Figure 2b). Nobiletin—at all concentrations—markedly reduced both early and late apoptosis. These results were consistent with the anti-apoptotic activity of nobiletin in endoplasmic reticulum stress-induced PC12 and I/R-exposed Kupffer cells [25].

Caspase-3 is a biomarker of oxidative-stress-stimulated cell death that has also been implicated in the final stage of apoptosis. The caspase-3 was obviously activated in the Aβ_25-35_-treated group (*p* < 0.001, Figure 2c). However, enhanced caspase-3 activity was dose-dependently decreased by nobiletin. Aβ-triggered apoptotic cell death is related to the reduced anti-apoptotic Bcl-2 protein and the increased pro-apoptotic molecule Bax expression. In a previous study, nobiletin displayed an anti-apoptosis effect by decreasing the ratio of Bcl-2/Bax expression in H_2_O_2_-stimulated HT22 cells [26].

### 3.3. Nobiletin Suppresses Aβ_25-35_-Induced Release of Inflammatory Markers

**Figure 3:**
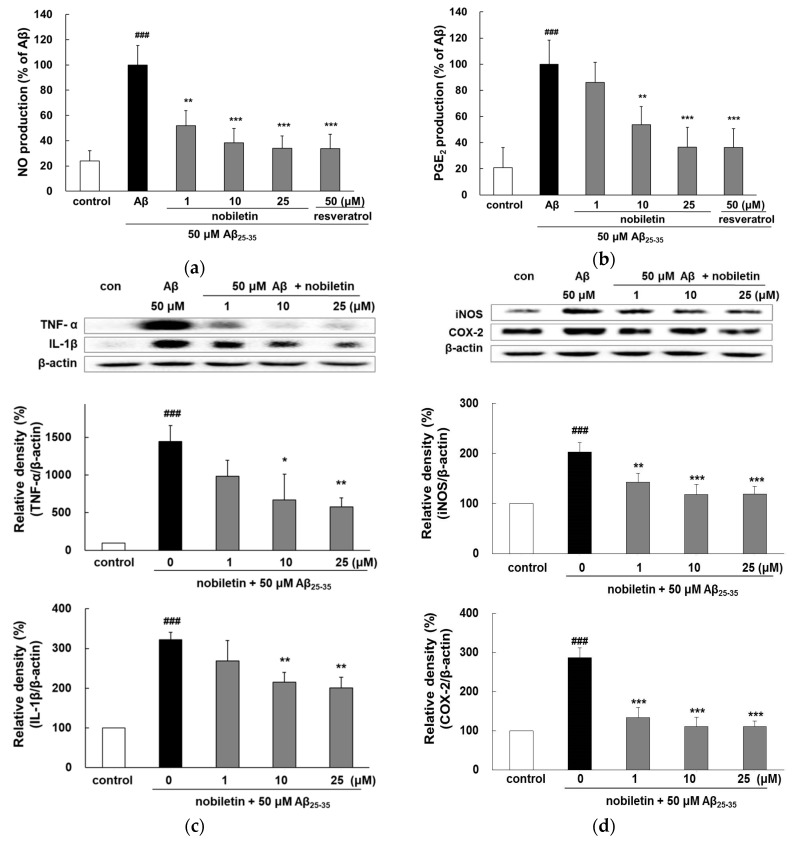
Inhibitory properties of nobiletin on Aβ_25-35_-mediated expression of (**a**) NO, (**b**) PGE_2_, (**c**) TNF-α and IL-β, and (**d**) iNOS and COX-2 in PC12 cells. The cells were pretreated with nobiletin for 1 h and then exposed to Aβ_25-35_ for 24 h. The culture supernatant was collected to evaluate the NO and PGE_2_ formation. Protein expression of TNF- α, IL-β, iNOS, and COX-2 was measured by Western blot analysis. The band intensities were quantified using Image J software and normalized to β-actin. ^###^ *p* < 0.001 vs. control. *** *p* < 0.001, ** *p* < 0.01, and * *p* < 0.05 vs. Aβ_25–35_.

Aβ_25-35_ exposure increases the release of NO and PGE_2_ by approximately 5-fold compared with the control (Figure 3a,b). Notably, treatment with 10 and 25 μM nobiletin exhibited similar activity as that of 50 μM resveratrol. Aβ_25-35_ treatment increased the expression level of pro-inflammatory cytokines, such as TNF-α and interleukin (IL)-1β, by 3-fold (Figure 3c), but the expressions were suppressed by 10 and 25 µM nobiletin.

As shown in Figure 3d, the level of iNOS stimulated by Aβ_25-35_ increased up to 202.5% ± 18.3% compared with the control (*p* < 0.001). However, nobiletin promptly inhibited the expression of iNOS protein. Notably, the highest concentration of nobiletin resulted in almost complete suppression of the enzyme production (118.3% ± 15.7%; *p* < 0.001). In parallel, Aβ_25-35_-mediated COX-2 expression was also markedly blocked by nobiletin.

Several studies have demonstrated that nobiletin possesses strong anti-inflammatory ability in lipopolysaccharide (LPS)-induced expression of pro-inflammatory cytokines in BV2 microglial cells [27,28,29]. A recent in vivo study suggested that the oral administration of nobiletin (100 mg/kg/day) for 6 weeks attenuated microglial activation and secretion of pro-inflammatory mediators, leading to the restoration of memory deficits in mice [30].

### 3.4. Nobiletin Regulates Aβ_25-35_-Induced NF-κB and MAPK Signaling Pathways

**Figure 4:**
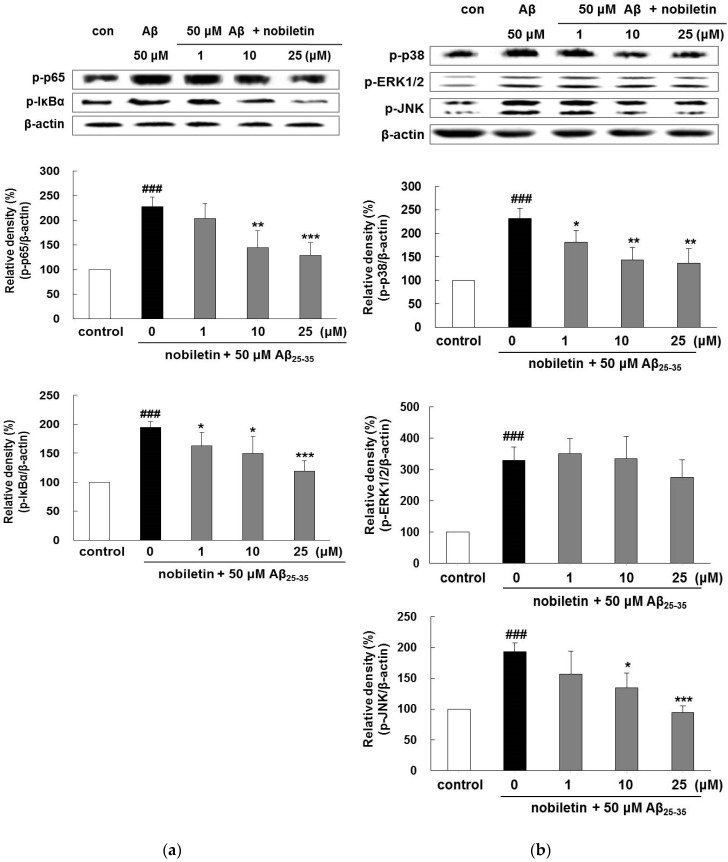
Inhibitory properties of nobiletin in the NF-κB/MAPKs pathway. Phosphorylation of (**a**) p65 and IκB-α, and (**b**) p38, ERK1/2, and JNK was examined by Western blot analysis. The cells were pretreated with nobiletin for 1 h and then exposed to Aβ_25-35_ for 4 h (p65 and IκB-α) or 1 h (p38, ERK1/2, and JNK). Quantification of band intensities was conducted using Image J software and normalized to β-actin. ^###^ *p* < 0.001 vs. control. *** *p* < 0.001, ** *p* < 0.01, and * *p* < 0.05 vs. Aβ_25–35_.

Aβ_25-35_ obviously elevated the phosphorylation of p65 and IκB-α by 227.4% ± 19.6% and 194.4% ± 10.1%, respectively. In contrast, pretreatment with nobiletin at 10 and 25 µM remarkably repressed p65 expression (Figure 4a). Moreover, in the case of IκB-α, all doses of nobiletin indicated a significant inhibitory effect in respect to Aβ_25-35_ treatment. 

As presented in Figure 4b, nobiletin suppressed Aβ_25-35_-evoked phosphorylation of p38 and JNK. In particular, the level of phosphorylated JNK was markedly suppressed when treated with 25 µM nobiletin (94.4% ±  9.8%, *p* < 0.001), indicating that the phosphorylation of MAPKs was closely related with the inhibitory property of nobiletin on Aβ_25-35_-evoked activation of p65 and IκB-α.

Previous research has reported that BACE1 promoter transactivation is modulated by the NF-κB signaling pathway, indicating that the suppression of NF-κB leads to inhibition of BACE1 activity [9]. In our previous study, we observed that nobiletin blocked BACE1 activity, suggesting that the compound might modulate BACE1 activation by suppressing the NF-κB signaling pathway [19]. Another study reported that nobiletin inhibited H_2_O_2_-evoked cell death in HT22 murine hippocampal cells, accompanied by decreased JNK and p38 phosphorylation [26]. In rat primary astrocytes, nobiletin suppressed the overexpression of iNOS and NO by the inhibition of the NF-κB/p38 MAPK pathways.

Several animal studies clearly demonstrated that nobiletin (10–50 mg/kg) improves memory in rats exposed to chronic intracerebroventricular infusion of Aβ_1-40_ [31]. In addition, daily supplementation of nobiletin (10 mg/kg) for 4 months significantly lowers both Aβ_1-40/42_ and amyloid plaques in 9-month-old APP-SL 7–8 Tg mice [17]. Recently, Nakajima et al. demonstrated that nobiletin (10 mg/kg) reduces tau phosphorylation in SAMP8 mice [23].

Bioavailability is an essential factor for the development of potential therapeutic agents. In addition, the anti-AD agents must penetrate the blood–brain barrier to attain sufficient concentration for the therapeutic application within the central nervous system. When nobiletin (50 mg/kg) was administered, the content of intact nobiletin was detected in rat brains within 1 h after dosing, suggesting that this compound can rapidly cross the blood–brain barrier and reach the brain. Furthermore, the concentration of nobiletin was 2.4-fold higher in the brain than in plasma. Interestingly, nobiletin was detected for up to 24 h in the brain, whereas in the plasma, it was observed up to 9 h, suggesting that elimination of this compound from the brain was slower in comparison to plasma [32]. Of note, nobiletin has no discernible toxicity in chronic treatments in mice and humans [33].

## 4. Conclusions

The novel results of the present study indicate that nobiletin, a natural compound in citrus peel, has a neuroprotective effect on Aβ_2__5-35_-induced cytotoxic damage of PC12 cells. The data may provide a preventive and/or therapeutic potential of nobiletin for degenerative disorders of the brain.
